# Congenital Diaphragmatic Hernia With Intra Thoracic Gastric Volvulus: A Rare, Life-Threatening Combination

**DOI:** 10.7759/cureus.38354

**Published:** 2023-04-30

**Authors:** Shishir Kumar, Rahul Saxena, Ranjan Kumar, Shivraj Chauhan, Kumar Diwakar

**Affiliations:** 1 Department of Surgery, Tata Main Hospital, Jamshedpur, IND; 2 Department of Surgery, Manipal Tata Medical College, Jamshedpur, IND; 3 Department of Pediatrics, Tata Main Hospital, Jamshedpur, IND

**Keywords:** life threatening, rare, pain abdomen, gastric volvulus, congenital diaphragmatic hernia

## Abstract

Congenital diaphragmatic hernia (CDH) is a known cause of secondary gastric volvulus (GV). Both entities are life-threatening, either alone or in exceedingly rare instances when they occur in combination. Here, we describe one such rare combination of CDH and secondary GV in a nine-year-old boy, who presented to us with recurrent episodes of abdominal pain. Urgent laparotomy was done after radiological evaluation (X-ray of chest and abdomen and contrast-enhanced CT chest and abdomen), which revealed mesenterico-axial volvulus of the stomach, secondary to an underlying diaphragmatic defect in the left hemidiaphragm, thus establishing the cause (diaphragmatic defect) and effect (GV), and resulting in a favourable outcome.

## Introduction

Gastric volvulus (GV) is a rare clinical event that can occur in both adult and pediatric patients [1]. It occurs when the stomach rotates at least 180 degrees along its transverse or longitudinal axis [2]. Primary GV results from laxity of ligamentous attachments [3]. When it occurs in the setting of underlying anatomical defects of the stomach, spleen, or diaphragm, it is called secondary GV [4]. More common is organo-axial volvulus where the stomach rotates around the pylorus and the gastroesophageal junction [5]. If the stomach rotates on a longitudinal line parallel to the gastrohepatic omentum, it is called mesentericoaxial volvulus [6]. Here, we describe a rare presentation of GV with congenital diaphragmatic hernia (CDH) in a nine-year-old boy, who presented to us with recurrent episodes of abdominal pain.

## Case presentation

**Figure 1:**
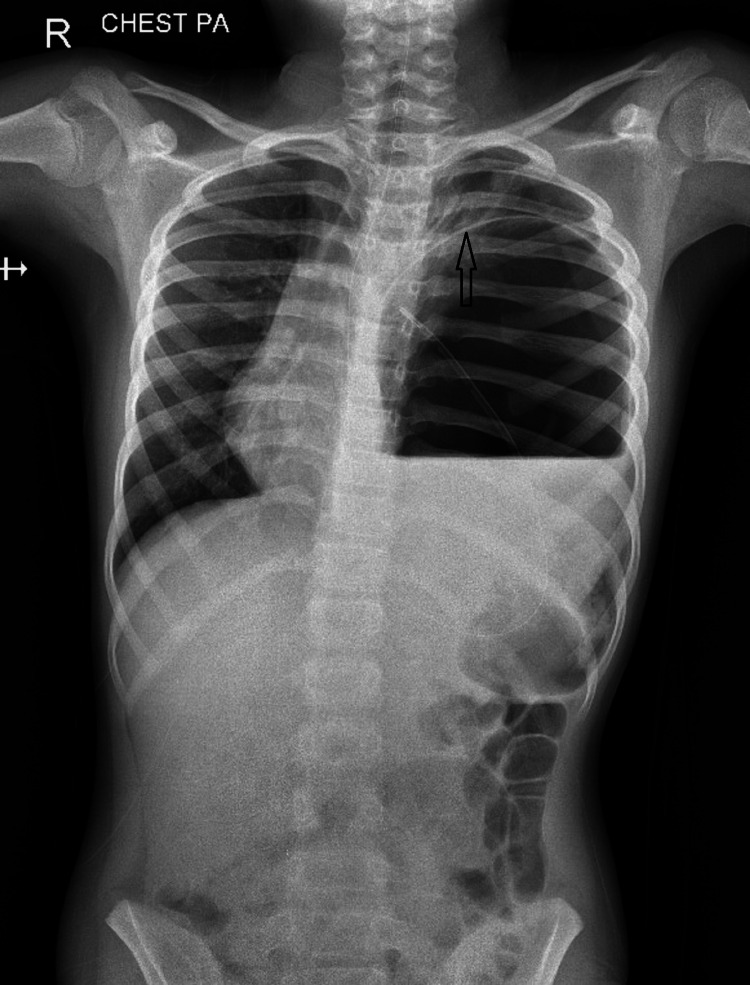
Air pocket in left hemithorax, with a thin rim separating it from lung parenchyma, with nasogastric tube in air pocket

A nine-year-old boy was admitted with acute onset abdominal pain of three hours duration, without any complaints of vomiting. He had a history of several episodes of similar pain in the preceding three months, with resolution on conservative management. There was no history of trauma, tuberculosis, gallstones, vomiting, fever, or weight loss. Weight and height were appropriate for age. The abdomen was soft on palpation, without any point tenderness, guarding or rigidity. Air entry on left lung field was restricted with hyper resonance on percussion. The boy had tachycardia (heart rate 124/minute), and tachypnea (Respiratory rate 30/minute). Blood examination showed haemoglobin (Hb) 10 g/dl with neutrophilic leukocytosis (total counts 17,000, Neutrophils 84%). Blood gas analysis showed normal lactate levels and no base deficits. X-ray of the chest and abdomen was suggestive of an air pocket in the left hemithorax with a thin rim separating it from lung parenchyma. Repeat X-ray after inserting a nasogastric tube did not show any significant change (Figure 1).

**Figure 2:**
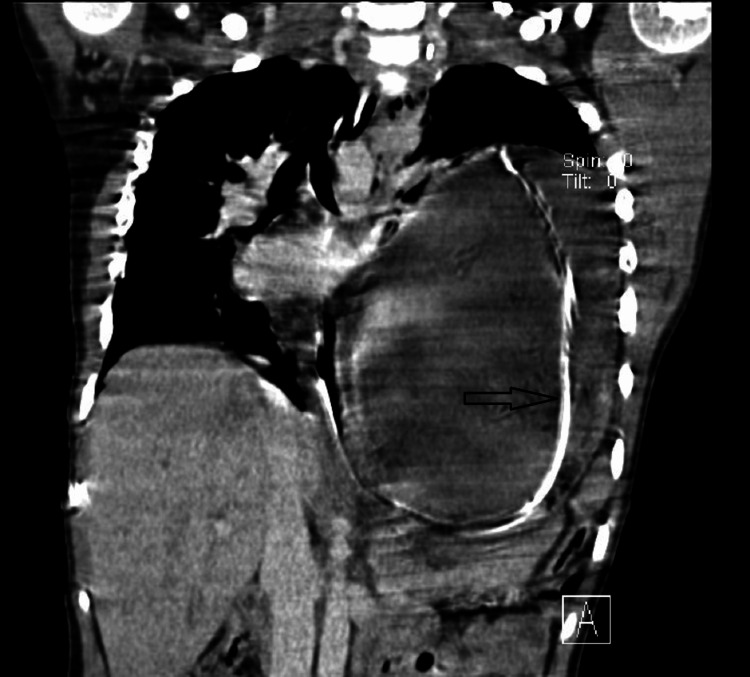
Herniated stomach in left hemithorax with nasogastric tube

**Figure 3:**
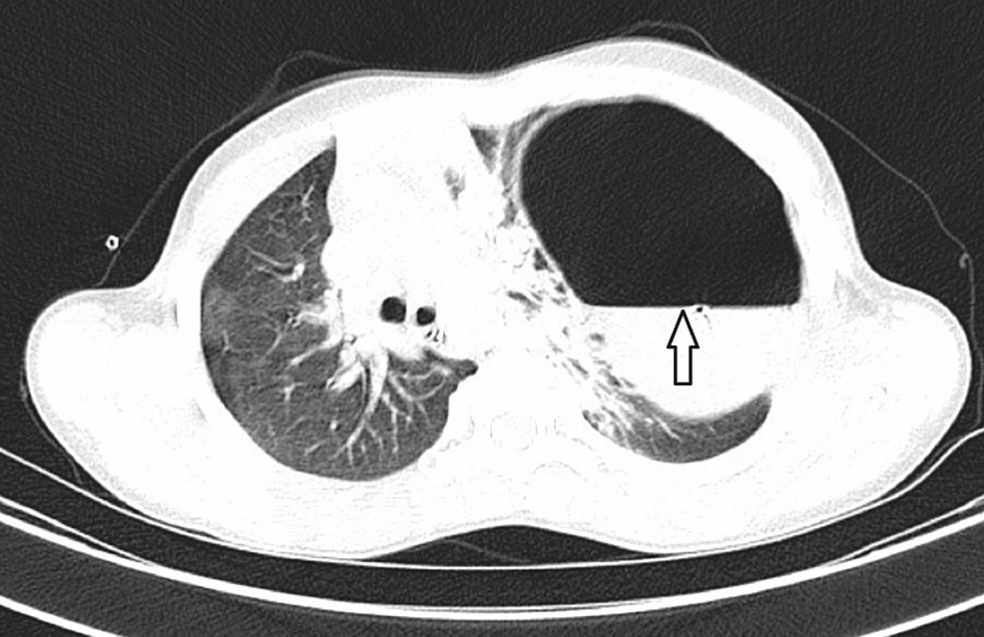
Cross-sectional CT view showing volvulus in herniated stomach

An urgent contrast-enhanced CT scan (CECT) of the chest and abdomen was done, which was suggestive of secondary GV with an underlying defect in the left hemidiaphragm (Figures 2, 3).

**Figure 4:**
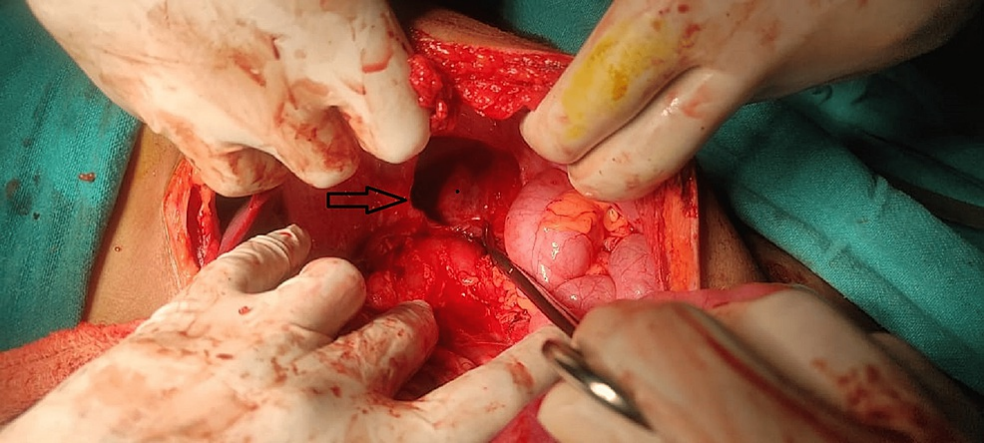
Defect in left hemi-diaphragm after reduction of herniated viscera

**Figure 5:**
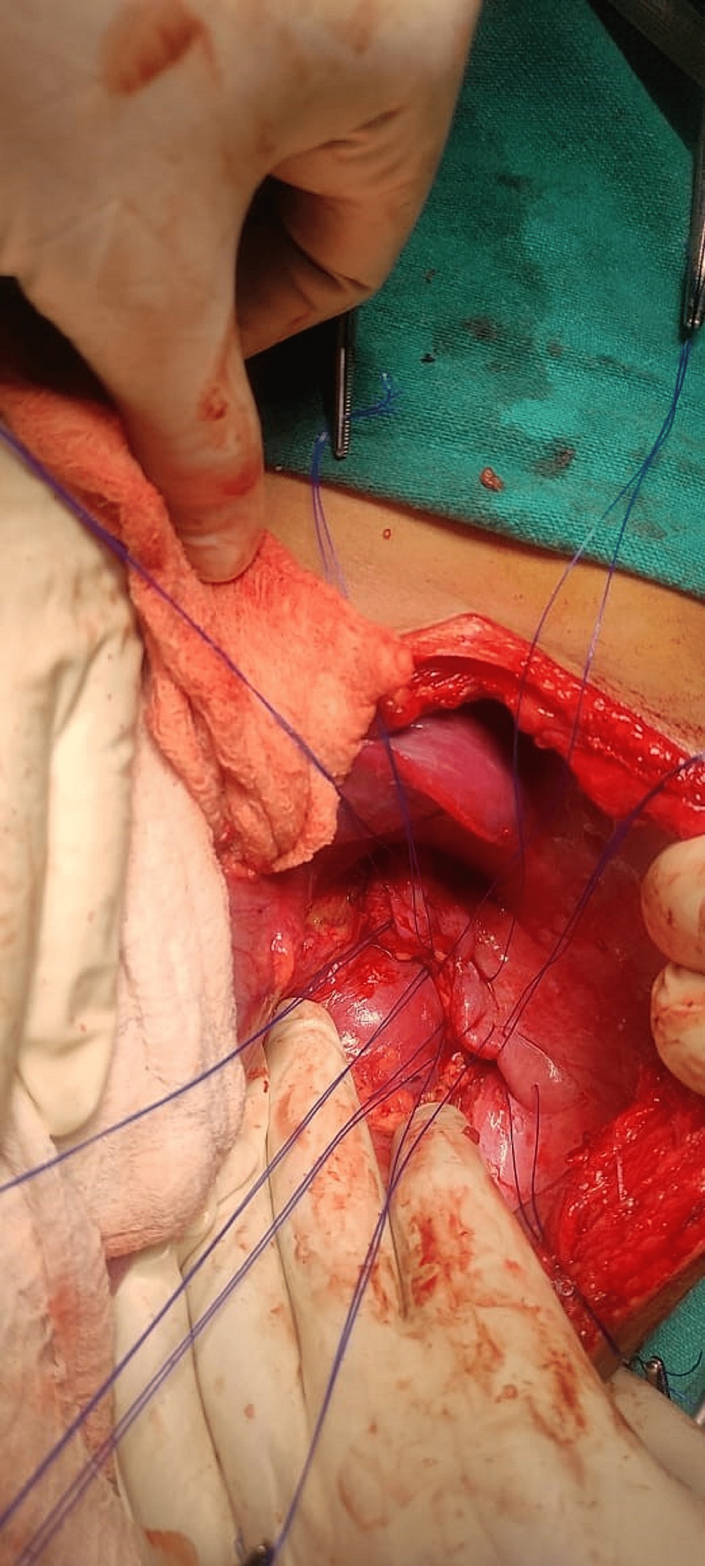
Closure of diaphragmatic defect by interrupted prolene sutures

Urgent abdominal exploration was planned after taking informed written consent from the parents. The abdominal cavity was explored through a supraumbilical midline incision. The whole of the stomach, the left half of transverse colon, and the spleen were reduced from the left thoracic cavity into the abdomen. Volvulus was reduced, and gastrostomy with anterior abdominal wall over an 8 Fr Foley catheter was performed as gastropexy. No gangrene/perforation was seen in the reduced stomach. The defect in the left hemidiaphragm was repaired with interrupted prolene sutures (Figures 4, 5). 

**Figure 6:**
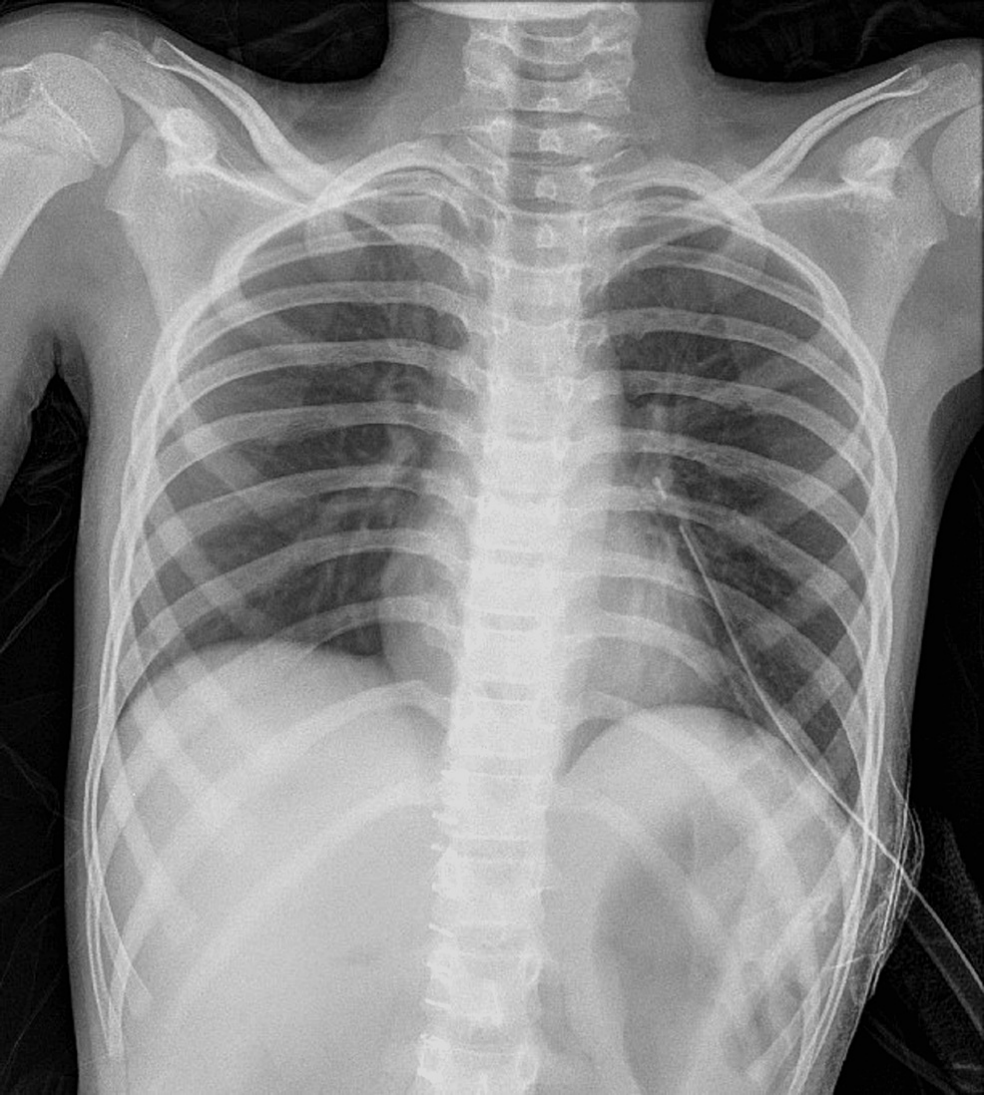
Full expansion of left lung, in postoperative period

An intercostal tube was placed in the left hemithorax. The postoperative period was uneventful, with a resumption of bowel activity on postoperative day 2. Full expansion of the left lung was seen in postoperative chest X-ray (Figure 6).

The intercostal tube was removed on the fifth postoperative day. The boy was discharged on the seventh postoperative day, and gastrostomy was removed after two weeks, on follow-up. At the time of writing this report, there have been two follow-up visits, one month apart, and the boy has been doing well.

## Discussion

GV is a surgical emergency, with a potential threat to life if not detected early enough and intervened. Diagnosis demands awareness of the entity on the part of the attending surgeon, meticulous clinical examination, and radiological assessment. In this case, the chest X-ray was initially interpreted as pneumothorax. But the history of multiple episodes of pain abdomen in the prior three months, along with normal oxygen saturation at room air ruled out pneumothorax as the final diagnosis. A provisional diagnosis of GV with an underlying defect of diaphragm was entertained, and subsequent CT scans and surgical exploration confirmed the findings.

This was a case of secondary GV due to the presence of an underlying defect in the diaphragm. The surgical principle of reduction of volvulus, decompression of stomach and fixation to the anterior parietal wall, and closure of underlying diaphragmatic defect were adhered to [7]. Initial decompression with nasogastric tube was also tried but failed. Attempts of gastric decompression by endoscopic aspiration and gastrostomy, and laparoscopic gastric fixation have also been described in the literature [8]. In our case, the volvulus being intra-thoracic, decompression by endoscopic decompression was ruled out as a measure of decompression. Upfront laparotomy helped in the salvage of the whole of the stomach, gastrostomy, and closure of the underlying diaphragmatic defect; thus Borchardt’s triad (retching, inability to pass gastric tube, and epigastric distension) has been a classical association with GV. This association may not always be seen in paediatric cases, as was in our case [9].

Barium meal or fluoroscopy have been described as investigative modalities in cases of GV, giving characteristic radiological signs [10]. In our case, the diagnosis could be made with clinical history and CT scans, beyond reasonable doubt, so upfront laparotomy was done to salvage the stomach.

GV, if not detected and intervened early, carries a mortality rate approaching 80% [11]. The main risk is gastric gangrene due to vascular compromise, necessitating total gastrectomy, and esophagojejunal anastomosis. In this case, prompt surgical intervention led to the salvage of the stomach before any vascular compromise could happen.

Multiple intermittent episodes of pain in the abdomen, in this case, could be explained by incarceration of herniated stomach through the diaphragmatic defect with spontaneous episodes of reduction of the herniated stomach, leading to the resolution of symptoms.

## Conclusions

GV is a surgical emergency, and correction of underlying anatomical defect is a vital step to prevent recurrence, and persistence of symptoms. Time is gold in such cases, and delay in intervention may lead to gastric resections, and major gastrointestinal reconstructions. Astute clinical examination and disease awareness helps in early diagnosis and prompt treatment.
